# Sustaining Effect of Intensive Nutritional Intervention Combined with Health Education on Dietary Behavior and Plasma Glucose in Type 2 Diabetes Mellitus Patients

**DOI:** 10.3390/nu8090560

**Published:** 2016-09-13

**Authors:** Rui Fan, Meihong Xu, Junbo Wang, Zhaofeng Zhang, Qihe Chen, Ye Li, Jiaojiao Gu, Xiaxia Cai, Qianying Guo, Lei Bao, Yong Li

**Affiliations:** Department of Nutrition and Food Hygiene, School of Public Health, Peking University, Beijing 100191, China; rfcaubj@126.com (R.F.); xumeihong@bjmu.edu.cn (M.X.); bmuwjbxy@bjmu.edu.cn (J.W.); zhangzhaofeng@126.com (Z.Z.); qiheyuntian@163.com (Q.C.); lydia30130@163.com (Y.L.); jiaojiaogu442@gmail.com (J.G.); shuiruoran8886@126.com (X.C.); mia1014@163.com (Q.G.); baolei6230@163.com (L.B.)

**Keywords:** nutritional intervention, health education, type 2 diabetes mellitus, diet, plasma glucose

## Abstract

Diabetes mellitus is very common in elderly Chinese individuals. Although nutritional intervention can provide a balanced diet, the sustaining effect on at-home dietary behavior and long-term plasma glucose control is not clear. Consequently, we conducted a long-term survey following one month of experiential nutritional intervention combined with health education. Based on the Dietary Guidelines for a Chinese Resident, we found that the food items met the recommended values, the percentages of energy provided from fat, protein, and carbohydrate were more reasonable after one year. The newly formed dietary patterns were “Healthy”, “Monotonous”, “Vegetarian”, “Japanese”, “Low energy”, and “Traditional” diets. The *2*h-PG of female participants as well as those favoring the “Japanese diet” decreased above 12 mmol/L. Participants who selected “Japanese” and “Healthy” diets showed an obvious reduction in FPG while the FPG of participants from Group A declined slightly. “Japanese” and “Healthy” diets also obtained the highest DDP scores, and thus can be considered suitable for T2DM treatment in China. The results of the newly formed dietary patterns, “Japanese” and “Healthy” diets, confirmed the profound efficacy of nutritional intervention combined with health education for improving dietary behavior and glycemic control although health education played a more important role. The present study is encouraging with regard to further exploration of comprehensive diabetes care.

## 1. Introduction

Diabetes mellitus (DM), one of the most serious chronic diseases in humans, affected approximately 347 million individuals worldwide in 2011 [1], and the incidence rate is still increasing sharply on the global scale [2]. DM has brought severe health risks for the entire world [3], particularly Type 2 diabetes mellitus (T2DM) with its associated cardiovascular complications being a global health problem and a sizeable economic burden [4]. DM is very common in the Chinese adult population and is currently rising in both incidence and prevalence [5]; the prevalence of diabetes is estimated to be 11.6% among adults [6], making China’s diabetic population one of the highest in the world [7]. The prevalence also increases with age, which is troublesome considering the rapid increase in China’s population [8] and proportion of older adults, currently 22.5% of Chinese citizens are >60 years old [6].

Disease self-management, support, and nutrition therapy are important components of diabetes care and necessary to ensure favorable outcomes in all people with DM [8]. Nutritional therapy has become the most reliable support regimen through which a diabetic patient can benefit [9]. Multiple studies have provided evidence that diabetes nutrition therapy is effective in improving glycemic control and other metabolic outcomes [10,11]. Nutrition management is a key component in the long-term health and quality of life of T2DM patients [12]. Nutritional intervention and education can also be provided as part of a comprehensive diabetes self-management and support program [13]. There is much evidence to support the positive effectiveness of self-management education with collaborative information intervention, lifestyle intervention, and skills teaching intervention among T2DM patients on diabetic control [14]. The dietary counseling related to the management of diabetes has been proved to improve patients’ nutritional status, clinical status, effectiveness of treatment, quality of life, daily functioning, and survival [15,16]. Although nutritional intervention and health education can provide balanced diets and good eating habits, any lengthy period of centralized intervention is highly challenging to conduct. Self-management is paramount in T2DM patients living at home, however, research is scarce regarding whether or not patients continue to maintain balanced dietary behavior in the long run after centralized intervention; the effect of different dietary patterns formed while living at home with regard to sustained, effective control of T2DM merits further research. Attention to food portions and weight management, combined with physical activity, can help to improve glycemic control. General guidelines include 50%–60% of daily energy requirements derived from carbohydrates, low-glycemic-index foods, foods containing cereal fiber, and a protein intake of least 0.86 g/kg/day. A nurses’ health study and the health professional’s follow-up study found an inverse relationship between cereal fiber intake and DM development [17]. Hence, whole-grain intake was recommended as an effective diet for T2DM patients. Although recent researchers have investigated the effect of nutritional intervention on diabetic patients, with some going so far as to test the effectiveness of oats and structured diets on glycemic control, the characteristic of different diets formed after intervention during home-living (and further, their varied influences on plasma glucose level) remain unclear.

In order to reflect the actual dietary behavior of T2DM patients living at home after centralized intervention, and further develop an effective protocol for comprehensive, sustained T2DM management, we conducted a randomized, single-blind, multi-arm parallel trial for older adults with T2DM at a community health center in Baotou City, Inner Mongolia, China (Trial registration: NCT01495052 at ClinicalTrials.gov) over the course of one month following a long follow-up survey. We hope that the results provide valuable information regarding long-term, comprehensive diabetes care.

## 2. Methods

### 2.1. Study Design and Participants

#### 2.1.1. Participants

Participants were recruited through newspaper and television advertisements. To be eligible for participation, individuals were required to be 50+ years old and have received a T2DM diagnosis from a licensed physician at least one year prior to the study. The exclusion criteria employed included any serious chronic complication of diabetes, the use of glucocorticoids or other drugs, and coincident participation in another intervention trial. All study procedures were followed in accordance with the ethical standards of the sponsoring institution and all participants provided written, informed consent.

#### 2.1.2. Study Design

The study was approved by the China–Japan Friendship Hospital of the Ministry of Health of the People’s Republic of China and was consistent with both the Declaration of Helsinki of 1975 as revised in 1983 and the guidelines of the center’s institutional review board.

##### Phase I (Intensive Experiential Management)

**Figure 1:**
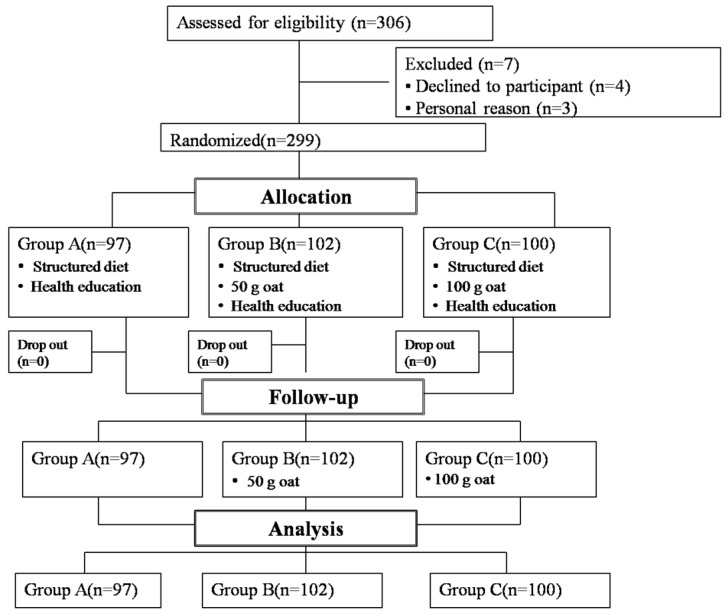
Flow diagram of subjects’ participation in the trial.

During the one-month centralized intervention portion of the study, all participants received uniformly arranged accommodation in a community health center in Baotou City under the supervision of strictly trained managers including nurses, diabetes educators, registered dietitians, and research staff. After a 1-week run-in period, the participants were randomized by a computer-generated number table and assigned accordingly into three groups. The “structured diet group” (Group A) was provided a standard diet, and “intervention oat groups” (Groups B and C) were provided the same diet coupled with a 50 or 100 g/day oat substitution for the equivalent grain (the Flow diagram of subjects’ participation is shown in Figure 1). The structured diet included foods typical of a standard diet in China, and was designed on a 7-day rotation according to the the Dietary Guidelines for a Chinese Resident [18] and the China Medical Nutrition Therapy Guideline for Diabetes 2010 [19], in which carbohydrate, protein, and fat provided 60%, 18%, and 22% of total energy, respectively (detailed information of the structured diet is shown in Table S1).

During the experiential intervention period, all participants were requested to continue their previously prescribed physical activity and medication habits. They also received 12 public lessons from professional nutritionists regarding nutrition and health including instructions for a healthy diet, physical exercise, and living habits, as well as elementary knowledge of diabetes (the contents of the public lessons are provided in Table S2 in Supplementary Materials). Each participant’s food intake and compliance to the assigned regimen were recorded daily by trained research staff. The technicians involved in the analyses and the statistician tasked with the preliminary assessment of the outcomes were blinded to the participants’ group allocations.

##### Phase II (Follow-up Survey)

After one-month centralized management, all participants returned home and 50 g and 100 g-oats were continuously provided to Group B and Group C by trained staff. Participants were required to record their daily dietary intake, any uncomfortable signs, and any changes in their medication. Scheduled clinical checks were performed every six months. The follow-up survey lasted for one year.

### 2.2. Outcome Measures

The primary outcome of the randomized controlled trial included the relative changes in hemoglobin A1c (HbA1c) and plasma glucose (PG). Assessments were conducted at baseline and at the one-year follow-up survey. During the one-month centralized management period, participants were required to refrain from medication and food for at least 12 h prior to blood collection. All examinations were performed by the same certified clinical staff (Inner Mongolia Medical College, Third Hospital, Baotou, China) at the same laboratory. Standardized questionnaires were used to obtain self-reported socioeconomic, demographic, cultural, and biological information.

### 2.3. Desirable Dietary Pattern (DDP) Score

N-DDP scores were utilized to evaluate the characteristic of dietary patterns [20]. The DDP score of a group of foods refers to its energy percentage multiplied by a certain rating, the rating defined by the nutrient content and nutritional quality of the given group of foods. The total score of a diet is the sum of the DDP scores of all groups of foods it contains. Table S3 shows the N-DDP score model utilized in the present study.

### 2.4. Statistical Analyses

All data was tested to ensure normal distribution in SPSS 18.0 (IBM, New York, NY, USA) prior to subsequent analysis (Skewness < 1, Kurtosis < 1).

A paired sample *t*-test was conducted to compare the changes before and after intervention within groups. Differences in response among groups were analyzed via one-way ANOVA, where results are presented as means and standard deviations. Duncan’s post hoc test was performed for multiple comparisons when changes were significantly different among groups.

The dietary results were analyzed via principal component analysis (PCA) with the correlation matrix as input [21]. The Kaiser-Meyer-Olkin (KMO) value was greater than 0.6, and results were considered significant at *p* < 0.001. The associations between interventional effects (including dietary patterns and PG) and sociodemographic factors were assessed according to the odds ratio (OR) with the logistic regression model [22]. The final model included those variables that showed a statistical significance of up to 5% (*p* < 0.05). All statistical procedures were performed using SPSS 18.0 software (IBM, New York, NY, USA).

## 3. Results

### 3.1. Dietary and Nutrient Intake

Of the total participants (299), 54% were female and 46% were male, 4.3% were illiterate, 9.8%, 26.2%, and 31.7% received primary, middle, and high school education, respectively, and 28% received college education or higher. A percentage of 25% were aged from 51 to 60 years, 62.2% from 61 to 70 years, and 12.2% were over 70 years old.

The daily intake of energy and nutrients was estimated based on 24-h dietary recall (Table 1). Intake of whole grains, eggs, vegetables, and beans were consistent with the Dietary Guidance for a Chinese Resident [18]. The diets at baseline showed low oat, fruit, fish, poultry, and dairy consumption among each group. The increased prevalence of these items in diets after the one-year intervention met the suggested values. Oil and salt intake at baseline were above suggested levels, but oil intake was reduced after intervention (*p* < 0.05). Oil, salt, and fish intake met the suggested values after the one-year intervention, as well, though dairy and fruit intake were still lower than the suggested values and red meat intake continued to exceed the recommended values [18].

Nutrient intakes are reported in Table 2. Energy intake decreased after the one-year intervention, though it was still relatively high. The intake of fats and carbohydrates decreased after intervention, while, fiber intake was higher than the baseline. The intake of all three energy-providing nutrients (carbohydrate, fat, and protein) were more reasonable after intervention [19].

### 3.2. PCA for the Dietary Factors

PCA was utilized to evaluate the refinement solution. The varimax rotation was applied for the final solution as it generated the strongest loadings on each of the final six factors (eigenvalues above 1) with the most meaningful interpretation. These six factors explained 62% of the total variance in the data; the each rotated dietary factors explained 13.7%, 10.7%, 10.5%, 9.5%, 9.4%, and 8.1% of the total variance, respectively (Table 3).

For the food items it made good theoretical sense to justify the inclusion of these factors, the final six dietary factors are summarized in Table 3. “Non-staple food” represents a high consumption of red meat, poultry, fruit and fish; “meal replacement food” characterizes significant consumption of whole grain and vegetable; “staple food” indicates a high intake of rice and bean; “soup food” includes salt and oats; “high protein food” refers to high intake of dairy and egg, and finally, “pastry food” is comprised of wheat and oil intake.

Fourteen food items were ultimately valid and foods with scores above 0.20 were lumped into the same category, where higher scores in the dietary category represented higher consumption of these foods. According to the three highest scores, the dietary patterns were defined as “Traditional”, “Vegetarian”, “Japanese”, “Low energy”, “Healthy”, and “Monotonous”, respectively (Table 4).

### 3.3. Evaluation of the Different Dietary Patterns (DDPs)

As shown in Table 5, the scores of six respective dietary patterns provided different levels of energy. Four were recognized as the most desirable: “Vegetarian”, “Japanese”, “Low energy”, and “Healthy” diets [20]. “Japanese” and “Healthy” diets, which contained large amounts of fruit, vegetable, milk, bean, fish, and tuber received the highest scores. These two diets were also relatively low-energy, and rich in protein, fiber, vitamins, and other bioactive compounds. “Traditional” and “Monotonous” diets provided more energy, partly due to their large amounts of meat and oil.

### 3.4. The Association between Dietary Patterns and Different Sociodemographic Variables

Table 6 shows the results of the multivariate regression analysis of variables associated with dietary patterns. Participants tended to certain dietary patterns dependent on their respective ages. Those younger than 70 were more likely to select “Traditional” diet rather than “Japanese” diet (OR = 0.032, 95% CI = 0.02 to 0.45) and “Healthy” diet (OR = 0.042, 95% CI = 0.01 to 0.45; OR = 0.061, 95% CI = 0.01 to 0.60). Education lever was a factor as well: participants with a high school education tended to prefer the “Traditional” diet (OR = 0.018, 95% CI = 0.04 to 0.72), while those with a lower level of education preferred the “Monotonous” diet (OR = 11.44, 95% CI = 1.04 to 125.2). Different interventional groups also made different choices: participants belonging to Group B preferred the “Low energy” diet over the “Traditional” diet (OR = 0. 13, 95% CI = 0.03 to 1.65).

### 3.5. The Effects of Nutritional Intervention Combined with Healthy Education on Plasma Glucose

**Figure 2:**
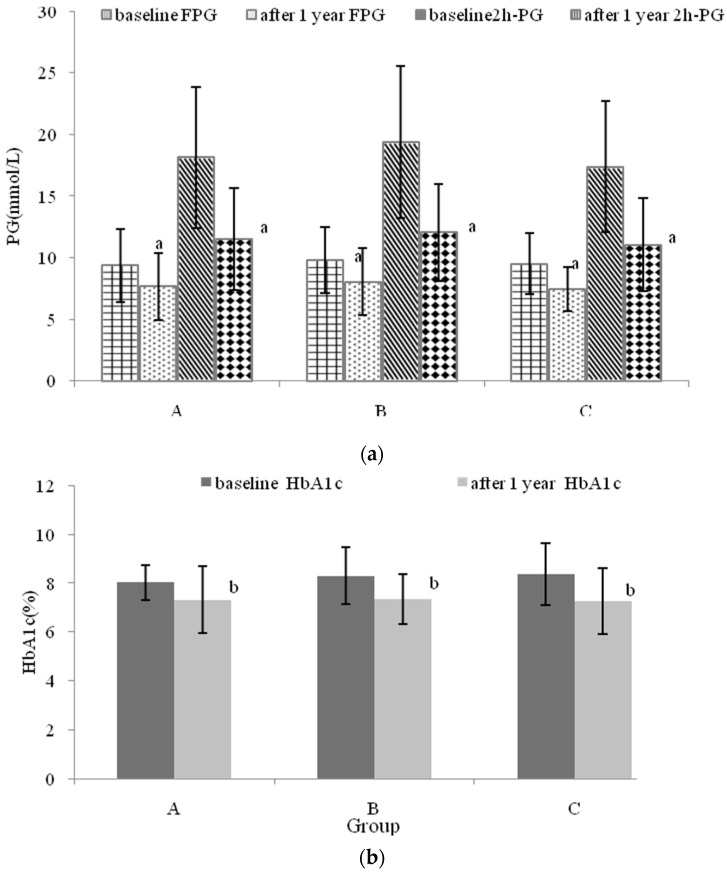

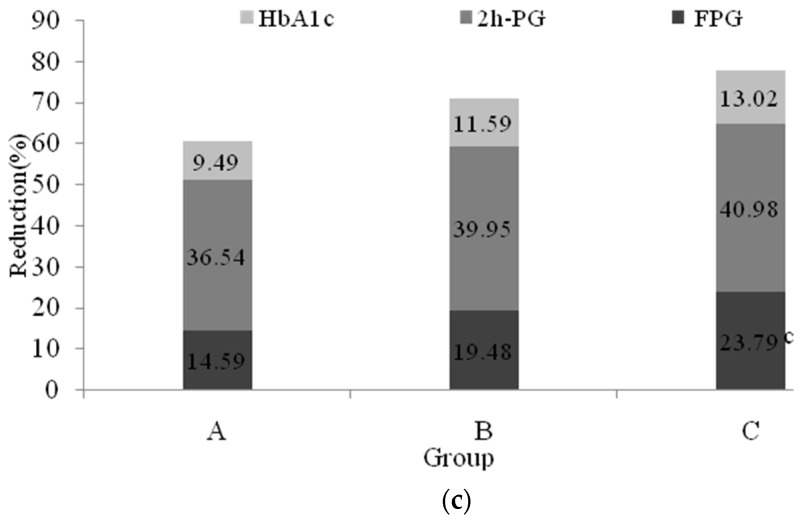
The 2h-plasma glucose (*2*h-PG) and fasting plasma glucose (FPG) of participants belonged to different groups. (**a**) the *2*h-PG and FPG value; (**b**) hemoglobin A1c (HbA1c) value; (**c**) the reduction of FPG, *2*h-PG and HbA1c. The results were expressed as mean (standard deviation). ^a^ compared with PG value at baseline (*p* < 0.05); ^b^ compared with HbA1c value at the baseline (*p* < 0.05); ^c^ compared with Group A at FPG reduction (*p* < 0.05).

One-year later, PG and HbA1c decreased significantly (*p* < 0.05) (Figure 2). FPG decreased significantly (*p* < 0.05) from 9.35 ± 3.1, 9.83 ± 2.6 and 9.48 ± 2.8 mmol/L in Group A, B, and C to 7.66 ± 2.5 (8.02 ± 1.9 and 7.44 ± 2.0 mmol/L in Group A, B, and C, while, *2*h-PG declined significantly (*p* < 0.05) from 18.14 ± 5.2, 19.36 ± 5.3 and 17.36 ± 5.1 mmol/L in Group A, B, and C to 11.51 ± 4.7, 12.05 ± 4.5 and 11.05 ± 4.3 mmol/L in Group A, B, and C, respectively. Also, HbA1c decreased obviously (*p* < 0.05) from 8.02% ± 0.93%, 8.30% ± 1.02% and 8.35% ± 1.04% in Group A, B, and C to 7.32% ± 1.24%, 7.34% ± 1.04% and 7.26% ± 1.30% in Group A, B, and C, respectively (Figure 2b). It was observed from Figure 2c that the reduction of FPG, *2*h-PG and HbA1c in Group A was smaller than that in Groups B and C after one year of intervention, the FPG of Group C decreased to a greater extent (23.79%) than it did in Group A (14.29%) (*p* < 0.05).

**Figure 3:**
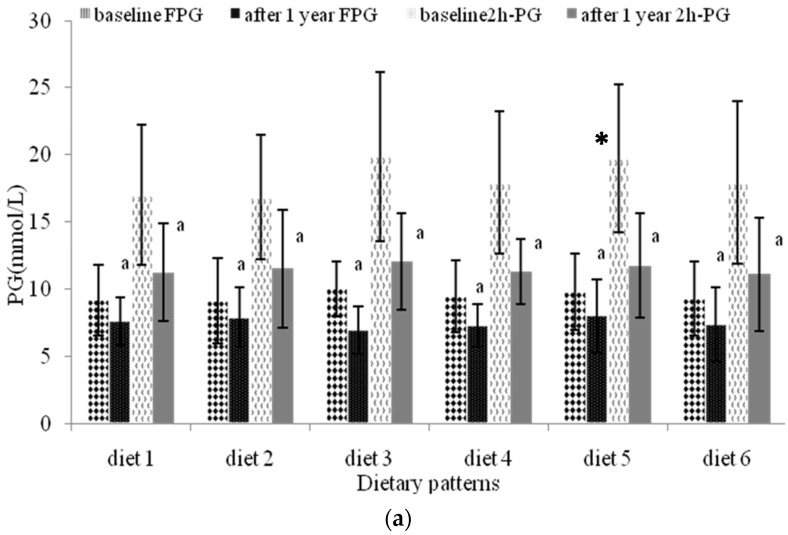

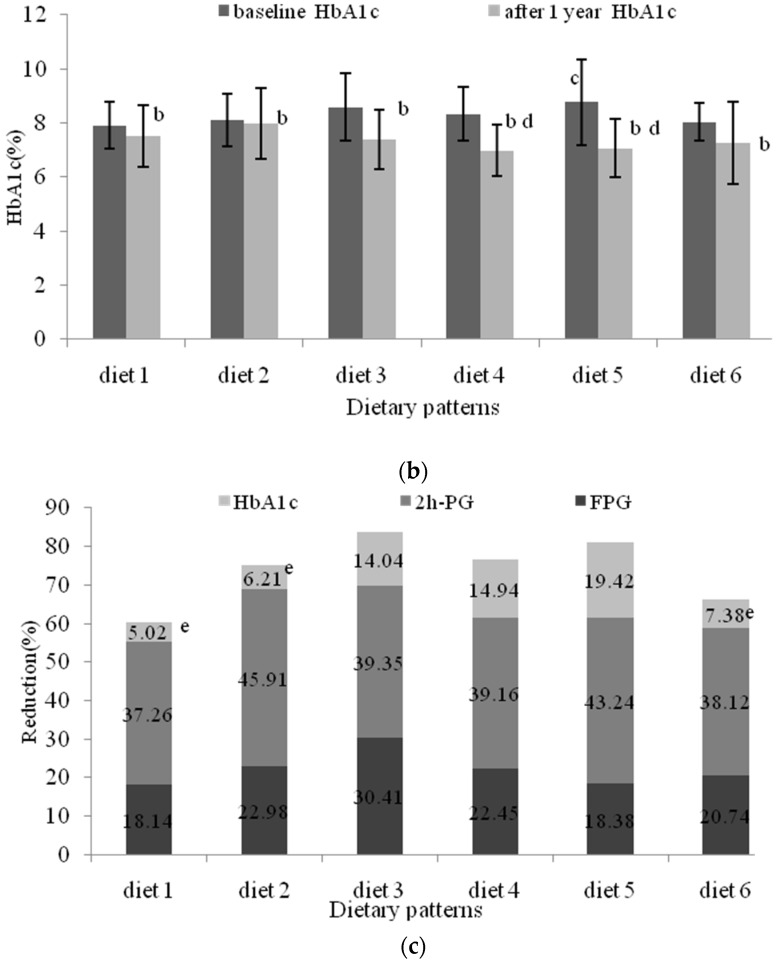
The *2*h-PG and FPG of participants selected different dietary patterns. (**a**) the *2*h-PG and FPG value; (**b**) HbA1c value; (**c**) the reduction of FPG, *2*h-PG and HbA1c. Diet 1, Traditional diet; Diet 2, Vegetarian diet; Diet 3, Japanese diet; Diet 4, Low energy diet; Diet 5, Healthy diet; Diet 6, Monotonous diet. The results were expressed as mean (standard deviation). ^a^ compared with PG value at baseline (*p* < 0.05); ^b^ compared with HbA1c value at baseline (*p* < 0.05); ^c^ HbA1c value at the baseline compared with the diet 1 (*p* < 0.05); ^d^ HbA1c value after 1 year was compared with the diet 2 (*p* < 0.05); ^e^ HbA1c value reduction was compared with the diet 5 (*p* < 0.05); * compared with *2*h-PG value of diet 1 at the baseline (*p* < 0.05).

Differences in the participants’ dietary patterns have an impact on their PG levels (Figure 3). FPG and *2*h-PG decreased significantly (*p* < 0.05) after the one-year intervention. Participants who selected “Japanese” diet presented the lowest FPG among the six patterns (*p* < 0.05). The *2*h-PG value at baseline for participants who selected the “Japanese” and “Healthy” diets were relatively high. “Healthy” diet yielded higher *2*h-PG than “Traditional ”diet (*p* < 0.05), while the new *2*h-PG values after the one-year intervention were close to normal. Figure 3b shows where the HbA1c values also significantly decreased after one year (*p* < 0.05). At baseline, the HbA1c value of “Healthy” diet was highest and was significantly lower than “Vegetarian” diet after one year (*p* < 0.05). Reduction of FPG, *2*h-PG, and HbA1c are shown in Figure 3c. The HbA1c reduction in “Healthy” diet was highest (19.42%)—considerably higher than “traditional diet” (5.02%) (*p* < 0.05) and “vegetarian diet” (6.21%) (*p* < 0.05), and “Vegetarian” diet resulted in the highest 2h-PG among all six diets (*p* < 0.05). FPG reduction was most obvious in “Japanese” diet.

Table 7 shows the results of the multivariate regression analysis for *2*h-PG reduction. Most notably the said reduction in *2*h-PG exceeded 12 mmol/L for female participants. The effects of all six dietary patterns on *2*h-PG were significant. The *2h*-PG for participants who selected “Japanese” diet decreased even exceeding 12 mmol/L after one-year intervention (OR = 0.09, 95% CI = 0.01 to 0.65).

Table 8 shows the results of the multivariate regression analysis for FPG reduction. We also observed distinctions among FPG reduction by age range. Compared to participants over 70 years old, those 51 to 60 years old showed no decrease in FPG after the one-year intervention (OR = 67, 31, 95% CI = 1.8 to 251.3). Participants in Group A showed a relatively slight decrease in FPG (OR = 7.50, 95% CI = 0.99 to 58.96). In addition, the effect of dietary patterns was also significant: compared to “Monotonous diet”, there was an obvious reduction of FPG in the range from 5 to 7 mmol/L observed in those participants who selected “Japanese” diet (OR = 0.08, 95% CI = 0.03 to 0.90) and “Healthy” diet (OR = 1.52, 95% CI = 0.15 to 0.94).

## 4. Discussion

The rising epidemic of obesity and increasing prevalence of diabetes worldwide has been partially ascribed to poor eating habits [23]. This study was conducted to elucidate the positive and sustained effect of intensive experiential management on dietary behavior and PG in T2DM patients.

The intake of oil, salt, and fat decreased after one year due to the one-month experiential intervention including a structured diet and health education, these results reflect the sustainable efficacy of intensive experiential management. The diets among the three groups showed the same intake in fiber, though oats intake varied from group to group. Importantly, the participants in Group A may have consumed increased levels of other cereals due to their improved health knowledge of nutrition through the health education they received, reflecting the importance of health education. This indeed helps to improve attitudes and encourage healthy dietary habits [4]. By virtue of receiving health education, the patients’ knowledge of appropriate self-care was expanded, this enabled them to more effectively manage their lives. In participants in this study, the improved dietary behavior led to effective FPG, *2*h-PG, and HbA1c control.

As oats intake increased, FPG, *2*h-PG, and HbA1c decreased considerably, which proved oat-based dietary supplementation helpful in the treatment of T2DM [2]. We observed alongside increased oats intake, the fiber supplied by oats correspondingly increased in Groups B and C, although total fiber intake remained insignificant among the three groups. There was also a positive linear correlation between PG and HbA1c reduction and oats (fiber) intake (*R*^2^ > 0.85) (data not shown), which suggests that the main contributor to PG and HbA1c was oats (fiber) consumption. These results are in accordance with the previous reports that oat fiber benefits low glycemic response [24,25,26,27]. Oats is an excellent dietary choice among the various whole grains available due to advantages in composition such as avenanthramides, or tocols, dietary fiber, especially β-glucan, and other nutrients [28,29,30]. The oats in this study, comprised of primary products peeled from naked oat, originates in northwest China (Patent Publication Number: CN101264459), and is rich in beneficial nutrients, (e.g., 3.05 g β-glucan per 100 g oats) [2]. We also observed a positive linear correlation between PG, HbA1c reduction and β-glucan intake (*R*^2^ > 0.90), indicating that β-glucan has a glucose-lowering effect because, oat β-glucan increases the insulin sensitivity index [31]. Oat β-glucan can also effectively improve the internal environment of the intestinal tract, increase intestinal Na^+^ K^+^-ATPase and Ca^2+^ Mg^2+^-ATPase activity [31], and may be a potential α-glycosidase inhibitor [32].

In this study, “Healthy” diet included staple food (rice) as well as non-staple food (fruit, vegetable), and also contained high quality protein not only from plant (beans) but also from animal (dairy, fish, and egg). However, it is worth mentioning that the so called “healthy diet” pattern had been composed of different foods in previous studies [22,23].

Although the diets in this study have varied in other reports, the “Healthy” diet is typically low in fat and rich in vitamins, minerals, and fiber, all of which are considered protective against non-transmissible chronic diseases [33]. The “Japanese” diet in this study contains fruit, vegetables, fish, red meat, beans, and rice, in similar proportions to a traditional Japanese diet [34]. This diet is associated with increased insulin sensitivity, and reduced risk of cardiovascular disease, hypertension, and cancer. Foods in the “Vegetarian” and “Japanese” diets are similar to those which comprise a traditional “Mediterranean diet” [35], long recognized as one of the most beneficial dietary patterns for DM patients across the globe [36].

Previous research has indicated that dietary behavior is dramatically influenced not only by sociodemographic factors, such as gender, age, education level, and marital status, but also by the quantity and quality of health-related information available [37]. It is thus reasonable that individuals with a higher degree of education will be more aware and keen on the nutritional quality of food, more likely to choose high-quality foods, and opt to form healthy diet patterns compared to less-educated individuals [38]. In the present study, we found that older, less-educated participants tended to prefer home-made and traditionally common foods, such as those found in the “Monotonous” diet and “Traditional” diet [39,40,41,42]. The significant differences in gender across the participants in this study were also relatively predictable. The *2*h-PG values in women decreased up to 12 mmol/L. We believe these differences are at least partially attributable to gender differences in health-consciousness. It has been well-documented that women pay more careful attention to health, beauty, and nutrition than men. Women also may be more likely than men to focus on changes in living quality, nutrition, and habits which led to the positive results [43,44,45]. They also may have been more likely to regularly and comprehensively self-report their dietary habits, benefiting the effectiveness and accuracy of their results [46,47]. This finding that women are more responsive is similar to other self-reports of nutrition labels in Canada [48].

Participants in this study between the ages of 51 and 70 years tended to select the “Traditional” diet. We believe this may be due to the prevalence of conservative thought in this population, as well as a general difficulty in simulating new concepts [38], together with a relatively low activity level. Their willingness to participate in health education and nutritional intervention is promising [49], however, in China, most older couples between the ages of 50 and 70 live together without children and are accustomed to a simple and frugal life, that may make it difficult to prepare rich, fresh, or varied foods. The same is especially true for elderly people who live alone [46], and may feel confined to a simple and familiar diet that is efficient and low in cost [50]. The majority of people over 70 years old living with and looked after by their children, however, may be assisted by them in dietary and lifestyle intervention. All in all, those participants between 51 to 60 years old showed poor glycemic control, and an increase in FPG after the one-year intervention.

Conversely, Group A participants (without oat intervention) showed relatively good FPG results. The main benefit was the effect of health education, which is in accordance with previous studies on the subject [51]. These healthy educational programs help patients and their families to improve their dietary and lifestyle habits by imparting self-care knowledge, health skills, and confidence, thus enabling them to take better control of their lives [52,53,54]. The importance of health education in terms of glycemic control has been well-established in a previous study; participants’ mean FPG decreased from 188.65 ± 71.45 mg/dL to 177.7 ± 66.11 mg/dL (*p* = 0.049) in a two-month educational intervention program [54].

We observed substantial reduction in FPG and *2*h-PG in participants who selected “Japanese” diet and “Healthy” diet. These two dietary patterns contained balanced nutrition and relative energy in the form of carbohydrates from sources such as fruits, vegetables, whole grains, beans, and milk. Also worthy of note is that “Japanese” and “Healthy” diets contained certain high-quality foods as in a “Mediterranean diet”, which has been associated with lower rates of cardiovascular disease, cancer, diabetes mellitus, hypertension, inflammation, Alzheimer’s disease, depression, and all-cause mortality [55,56,57,58,59]. The “Japanese” and “Healthy” diets prescribed here can, to this effect, be reasonably prescribed to aid in glycemic control.

The “Healthy” diet was the most often selected by participants in this study, which implies that the one-year nutritional intervention was quite effective in tandem with health education. The diet with the greatest benefit for diabetes sufferers, the “Japanese” diet, was chosen slightly less often, possibly suggesting certain limitations in the effectiveness of the education regimen. In addition, FPG and *2*h-PG decreased significantly after one year, but did not quite reach the target values, this may suggest further limitations, but also may imply that the intervention and education period was too short to be fully effective. Further, our sample was composed of Chinese individuals with T2DM, and therefore the results we derived might not apply to populations in other countries with other dietary patterns. Additionally, we did not determine whether self-reporting a specific behavior (e.g., foods consumed at breakfast, snacks, and beverage) was influential in changing the said behavior [60].

## 5. Conclusions

The results of this study demonstrated that participants did indeed improve their diet after intervention; FPG, *2*h-PG, and HbA1c decreased, which indicated the feasibility and sustainable efficacy of intensively experiential management with nutritional intervention, coupled with health education, for the elderly in China with T2DM. Undoubtedly, health education plays a particularly important role. Moreover, oat intake showed the obvious effect for hypoglycemics, the formed “Japanese” and “Healthy” diets could be suitable for T2DM, treatment due to the higher DDP scores and hypoglycemic effects.

## Figures and Tables

**Table 1:** Food items in each food category during pre- to post-intervention among the different groups.

	Intervention					
	Group A		Group B		Group C	
Food Item (g/day)	Baseline	Post-Intervention	Baseline	Post-Intervention	Baseline	Post-Intervention
Oil	30.54 (16.4)	22.31 (8.6) ^a^	29.11 (16.4)	24.07 (10.9)	29.07 (11.3)	24.51 (8.6)
Salt	6.79 (3.8)	4.58 (1.8)	7.07 (3.5)	5.87 (0.9)	6.31 (3.0)	6.10 (1.3)
Rice	107.06 (88.9)	135.30 (64.8)	127.83 (66.2)	131.13 (70.9)	113.12 (89.7)	136.67 (66.6)
Wheat	110.75 (78.8)	132.08 (84.5)	115.98 (99.3)	142.83 (86.3)	119.46 (58.6)	114.08 (49.9)
Whole grain	84.49 (60.1)	86.04 (59.0)	90.4 (69.1)	74.08 (34.3)	87.33 (57.2)	89.28 (51.3)
Vegetable	514.30 (265.6)	496.39 (74.4)	452.78 (159.0)	505.28 (185.8)	501.58 (356.2)	566.00 (234.0)
Fruit	142.20 (123.5)	157.1 (98.6)	105.38 (78.2)	128.62 (70.1)	153.32 (70.2)	188.00 (70.2)
Red meat	68.63 (38.4)	71.08 (48.5)	58.86 (25.4)	66.52 (39.5)	62.33 (45.5)	62.35 (30.4)
Poultry	21.96 (20.3)	56.00 (41.6)	21.85 (11.3)	49.75 (23.8)	22.44 (22.0)	54.88 (24.8)
Fish	39.43 (22.4)	64.13 (42.3)	54.65 (47.7)	66.81 (36.9)	52.75 (44.8)	75.00 (53.6)
Egg	50.71 (24.2)	60.75 (31.7)	59.93 (32.0)	50.18 (23.1)	64.34 (33.5)	58.33 (21.7)
Dairy	187.99 (112.0)	188.85 (94.5)	153.9 (86.7)	211.42 (58.4)	200.83 (81.2)	218.67 (68.5)
Bean	52.83 (42.5)	54.9 (35.7)	45.61 (35.7)	47.68 (26.3)	47.29 (33.6)	48.78 (30.4)
Oat	0	0	0	50 ^a,b,c^	0	100 ^a,b,d^

The results are expressed as mean (standard deviation). ^a^ The intakes of post-intervention were compared with the values at baseline in the same group (*p* < 0.05); ^b^ The intakes of post-intervention were compared with Group A values for the same period; ^c^ The intakes of post-intervention were compared with Group C values for the same period; ^d^ The intakes of post-intervention were compared with Group B values for the same period.

**Table 2:** The intake of nutrient during pre- to post-intervention among the different groups.

	Intervention					
	Group A		Group B		Group C	
Nutrition	Baseline	Post-Intervention	Baseline	Post-Intervention	Baseline	Post-Intervention
Energy (kcal/day)	2596.08 (964.9)	2480.00 (776.8)	2320.00 (886.0)	2225.00 (475.9)	2410.50 (709.5)	2315.10 (314.8)
Protein (g/day)	90.61 (30.5)	96.70 (32.1)	81.53 (39.1)	85.04 (25.8)	81.45 (23.6)	99.32 (25.4)
Protein energize	14.18%	15.80%	14.20%	15.51%	13.70%	17.40%
Fat (g/day)	78.51 (30.5)	72.24 (24.6)	75.87 (24.9)	66.89 (17.4)	70.56 (20.5)	68.93 (14.2)
Fat energize	27.47%	27.45%	29.7%	27.30%	26.60%	27.00%
Carbohydrate (g/day)	363.54 (135.6)	355.61 (104.4)	347.41 (118.8)	338.81 (75.8)	364.57 (120.2)	347.00 (54.1)
Carbohydrate energize	56.90%	58.10%	60.80%	61.20%	61.45%	60.90%
Total fiber (g/day)	39.90 (12.3)	41.39 (12.2)	38.54 (21.4)	41.40 (7.6)	38.86 (9.9)	44.85 (14.9)
Fiber from oats (g/day)	0	0	0	2.65 ^a,b,c^	0	5.3 ^a,b,d^

The results are expressed as mean (standard deviation); ^a^ The intake of post-intervention were compared with that of pre-intervention in the same group (*p* < 0.05); ^b^ The intake of post-intervention were compared with Group A values for the same period; ^c^ The intake of post-intervention were compared with Group C values for the same period; ^d^ The intake of post-intervention were compared with Group B values for the same period.

**Table 3:** Rotated coefficients for a 6-factor solution using principal component analysis and varimax rotation.

Food Item	Factors					
	Factor 1	Factor 2	Factor 3	Factor 4	Factor 5	Factor 6
	Non-Staple Food	Meal Replacement Food	Staple Food	Soup Food	High Protein Food	Pastry Food
Red meat	0.776	0.016	0.069	0.053	0.167	−0.222
Poultry	0.727	−0.126	0.201	−0.142	0.118	0.170
Fruit	0.581	0.330	−0.260	−0.077	−0.261	−0.071
Fish	0.481	−0.027	0.447	0.132	0.305	0.182
Whole Grain	0.004	0.754	0.113	−0.180	−0.043	0.084
Vegetable	−0.066	0.654	−0.105	0.311	0.271	0.130
Rice	−0.040	0.029	0.768	0.163	−0.321	−0.178
Bean	0.137	0.072	0.657	−0.093	0.217	0.105
Salt	0.138	0.228	0.238	0.741	−0.068	−0.187
Oats	−0.144	−0.151	−0.091	0.719	−0.012	0.105
Dairy	0.003	0.007	0.042	−0.157	0.752	−0.190
Egg	0.335	0.138	−0.013	0.141	0.558	0.146
Oil	0.048	0.201	0.075	0.025	−0.052	0.782
Wheat	0.203	0.499	0.172	0.116	0.023	−0.538

**Table 4:** Distribution of factorial loads of dietary patterns.

Dietary Patterns					
Pattern 1	Pattern 2	Pattern 3	Pattern 4	Pattern 5	Pattern 6
Traditional Diet	Vegetarian Diet	Japanese Diet	Low Energy Diet	Healthy Diet	Monotonous Diet
Red meat	Fruit	Red meat	Vegetable	Fruit	Red meat
Poultry	Whole Grain	Fruit	Salt	Fish	Oil
Fruit	Vegetable	Fish	Oats	Vegetable	Wheat
Fish	Salt	Rice		Rice	
Egg	Oil	Bean		Bean	
Wheat	Wheat	Salt		Dairy	
		Vegetable		Egg	

**Table 5:** Evaluation of the different dietary patterns (DDPs).

Diet	Score	Food Items (g/day)	Energy (kcal)
Traditional diet	79.76	cereal: 335, tuber: 65, meat: 274, fish: 96, egg: 51, milk: 188, bean: 34, vegetable: 528, fruit: 214, oil: 23	2556.6
Vegetarian diet	81.08	cereal: 392, tuber: 74, meat: 140, fish: 71, egg: 40, milk: 176, bean: 39, vegetable: 599, fruit: 200, oil: 23	2170.5
Japanese diet	82.52	cereal: 368, tuber: 53, meat: 164, fish: 94, egg: 41, milk: 163, bean: 58, vegetable: 428, fruit: 122, oil: 27	2236.4
Low energy diet	81.03	cereal: 310, tuber: 67, meat: 149, fish: 67, egg: 45, milk: 150, bean: 25, vegetable: 545, fruit: 127, oil: 25	2114.1
Healthy diet	81.27	cereal: 282, tuber: 79, meat: 171, fish: 71, egg: 58, milk: 224, bean: 41, vegetable: 642, fruit: 124, oil: 23	2241.0
Monotonous diet	78.08	cereal: 329, tuber: 69, meat: 155, fish: 62, egg: 49, milk: 165, bean: 34, vegetable: 559, fruit: 156, oil: 42	2403.0

**Table 6:** Analysis of the associations between different dietary patterns and variables.

Variables	Dietary Patterns																												
	Pattern 2 ^a^ Vegetarian Diet						Pattern 3 Japanese Diet						Pattern 4 Low Energy Diet						Pattern 5 Healthy Diet						Pattern 6 Monotonous Diet				
	*p*		OR		95% CI		*p*		OR		95% CI		*p*		OR		95% CI		*p*		OR		95% CI		*p*		OR	95% CI	
Gender																													
Female	0.782		1.19		0.35–3.94		0.752		1.22		0.36–4.10		0.988		1.01		0.29–3.51		0.300		1.82		0.59–5.69		0.430		1.63	0.48–5.48	
Male	1 ^b^					1								1				1						1					
Age (year)																													
51–60	0.528		0.38		0.02–7.73		0.011		0.032		0.02–0.45		0.155		0.14		0.01–2.13		0.009		0.042		0.01–0.45		0.428		0.29	0.01–6.08	
61–69	0.619		0.47		0.02–9.04		0.135		0.164		0.15–1.76		0.617		0.52		0.04–6.61		0.017		0.061		0.01–0.60		0.815		0.70	0.04–13.21	
>70	1 ^c^					1								1				1						1					
Education																													
Illiteracy	0.504		2.54		0.16–39.26		0.812		0.68		0.03–16.39		0.986		0.01		0.01–0.04		0.413		0.27		0.01–6.15		0.246		7.19	0.26–201.7	
Primary school	0.892		0.87		0.12–6.50		0.846		0.80		0.09–7.24		0.325		0.25		0.02–3.90		0.113		0.19		0.02–1.48		0.230		5.24	0.36–78.53	
Middle school	0.643		0.66		0.12–3.73		0.721		1.34		0.26–6.80		0.777		0.79		0.15–4.10		0.089		0.29		0.06–1.22		0.046		11.44	1.04–125.2	
High school	0.406		0.51		0.10–2.52		0.821		0.84		0.18–4.00		0.300		0.42		0.08–2.15		0.015		0.18		0.04–0.72		0.192		4.77	0.46–49.90	
>College	1 ^d^					1								1				1						1					
Group																													
Group A	0.409		1.98		0.39–10.04		0.492		0.59		0.13–2.65		0.970		0.01		0.01–0.09		0.458		1.75		0.40–7.68		0.407		0.513	0.11–2.49	
Group B	0.253		2.68		0.49–14.58		0.663		1.39		0.32–6.00		0.013		0.13		0.03–1.65		0.179		2.80		0.62–12.58		0.160		2.88	0.66–12.61	
Group C	1 ^e^					1								1				1						1					

^a^ Comparative reference group: Diet 1(Traditional diet); ^b^ Analysis adjusted for male; ^c^ Analysis adjusted for those older than 70 years old; ^d^ Analysis adjusted for those received college or higher education; ^e^ Analysis adjusted for Group C.

**Table 7:** Analysis of the associations between *2*h-PG reduction and different variables.

Varibles	*2*h-PG Reduction Level ^a^																																										
	Level ^b^ 0								Level 1									Level 2											Level 3										Level 4				
	*p*	OR			95% CI				*p*			OR			95% CI			*p*				OR			95% CI				*p*			OR		95% CI					*p*		OR	95% CI	
Gender																																											
Female	0.017	0.20			0.05–0.75				0.049			0.29			0.09–0.99			0.042				0.33			0.12–0.96				0.020			0.14		0.04–0.49					0.96		0.97	0.25–3.77	
Male	1 ^c^									1									1									1									1						
Age (year)																																											
51–60	0.207	5.62			0.29–81.78				0.415			0.42			0.05–3.44			0.551				1.84			0.25–13.78				0.681			1.58		0.18–13.86					0.681		0.65	0.08–5.00	
61–69	0.732	1.54			0.13–18.27				0.345			0.43			0.07–2.48			0.931				1.08			0.18–6.44				0.817			0.80		0.13–5.08					0.226		0.34	0.06–1.94	
>70	1 ^d^							1												1							1											1					
Education																																											
Illiteracy	0.460	3.52			0.12–99.51				0.999			0.01			0.01–0.56			0.257				4.26			0.28–64.93				0.277			5.16		0.27–99.47					0.110		0.01	0.01–0.09	
Primary school	0.998	0.01			0.01–0.12				0.983			1.02			0.12–8.59			0.759				1.29			0.25–6.72				0.542			1.81		0.27–12.28					0.998		0.01	0.01–0.07	
Middle school	0.651	1.49			0.26–8.52				0.437			1.81			0.40–8.15			0.548				0.65			0.16–2.64				0.970			1.03		0.22–4.71					0.110		3.89	0.73–22.60	
High school	0.276	2.62			0.46–14.84				0.317			2.23			0.46–10.77			0.248				2.16			0.58–8.00				0.993			1.00		0.19–5.36					0.126		3.76	0.69–20.53	
>College	1 ^e^									1									1									1									1						
Group																																											
Group A	0.857	1.17			0.22–6.34				0.125			3.83			0.69–21.37			0.222				2.41			0.59–9.93				0.480			1.77		0.36–8.67					0.357		2.28	0.39–13.21	
Group B	0.476	0.54			0.10–2.99				0.576			1.60			0.31–8.43			0.579				1.45			0.39–5.32				0.770			0.80		0.16–3.66					0.390		2.07	0.39–10.83	
Group C	1 ^f^									1									1									1									1						
Diet																																											
Diet 1	0.327	0.29			0.03–3.38				0.950			0.93			0.10–8.50			0.189				0.32			0.06–1.76				0.191			0.25		0.03–2.01					0.426		0.35	0.03–4.56	
Diet 2	0.211	5.35			0.39–73.90				0.487			2.60			0.18–38.46			0.723				0.67			0.07–6.18				0.854			0.78		0.06–10.75					0.508		2.68	0.14–49.83	
Diet 3	0.512	0.45			0.04–5.00				0.145			0.11			0.01–2.11			0.017				0.09			0.01–0.65				0.146			0.19		0.02–1.76					0.988		1.02	0.10–9.91	
Diet 4	0.351	0.28			0.02–4.00				0.800			1.37			0.12–15.23			0.341				0.40			0.06–2.66				0.204			0.22		0.02–2.68					0.368		0.25	0.01–5.06	
Diet 5	0.552	0.45			0.03–6.20				0.725			0.65			0.06–7.12			0.078				0.18			0.03–1.21				0.607			0.58		0.07–4.63					0.757		1.45	0.14–15.17	
Diet 6	1 ^g^									1									1									1									1						

Diet 1, Traditional diet; Diet 2, Vegetarian diet; Diet 3, Japanese diet; Diet 4, Low energy diet; Diet 5, Healthy diet; Diet 6, Monotonous diet. ^a^ *2*h-PG increasing refers to level 0, *2*h-PG reduction between 0 and 3 mmol/L refers to level 1, reduction between 3.1 and 6 mmol/L refers to level 2, reduction between 6.1 and 9 mmol/L refers to level 3, reduction between 9.1 and 12 mmol/L refers to level 4, reduction > 12 mmol/L refers to level 5; ^b^ Comparative reference level: level 5.; ^c^ Analysis adjusted for male; ^d^ Analysis adjusted for those older than 70 years old; ^e^ Analysis adjusted for those who received college or higher education; ^f^ Analysis adjusted for Group C; ^g^ Analysis adjusted for Diet 6.

**Table 8:** Analysis of the associations between FPG declined and different variables.

Varibles	FPG Reduction Level ^a^																																										
	Level ^b^ 0								Level 1									Level 2											Level 3										Level 5				
	*p*	OR			95% CI				*p*			OR			95% CI			*p*				OR			95% CI				*p*			OR		95% CI					*p*		OR	95% CI	
Gender																																											
Female	0.232	0.35			0.06–1.97				0.250			0.37			0.07–2.02			0.240				0.37			0.07–1.96				0.561			0.59		0.10–3.45					0.276		4.54	0.29–69.11	
Male	1 ^ c^									1									1									1									1						
Age (year)																																											
51–60	0.023	67.31			1.80–251.3				0.392			4.04			0.16–98.90			0.057				20.29			0.92–448.3				0.197			8.95		0.32–250.0					0.182		16.31	0.27–982.7	
61–69	0.069	13.75			0.80–232.5				0.744			1.43			0.15–13.75			0.310				3.08			0.35–26.84				0.226			4.13		0.42–41.06					0.388		4.34	0.15–127.8	
>70	1 ^d^							1												1							1											1					
Education																																											
Illiteracy	0.994	23.52			0.01–0.51				1.000			2.50			0.01–0.06			0.994				11.2			0.08–9.93				0.993			25.16		0.07–19.47					0.994		14.85	0.01–9.09	
Primary school	0.715	1.70			0.10–29.76				0.746			1.59			0.10–26.36			0.850				0.08			0.08–20.17				0.301			5.25		0.23–119.1					0.816		1.52	0.05–51.18	
Middle school	0.830	1.26			0.15–10.88				0.852			1.22			0.15–9.60			0.991				0.13			0.13–7.50				0.074			8.08		0.82–79.91					0.288		4.05	0.31–53.62	
High school	0.605	1.78			0.20–15.87				0.960			0.95			0.11–8.42			0.578				0.23			0.23–13.95				0.103			7.11		0.67–75.08					0.986		23.83	0.69–120.5	
>College	1 ^e^									1									1									1									1						
Group																																											
Group A	0.593	1.72			0.23–13.10				0.05			7.50			0.99–58.96			0.149				4.07			0.60–27.41				0.947			1.08		0.12–9.75					0.771		1.53	0.09–27.00	
Group B	0.427	2.18			0.32–14.95				0.27			2.99			0.43–20.79			0.141				3.86			0.64–24.32				0.178			3.80		0.54–26.44					0.360		3.49	0.24–50.49	
Group C	1 ^f^									1									1									1									1						
Diet																																											
Diet 1	0.759	1.52			0.11–21.65				0.736			0.66			0.06–7.23			0.706				0.64			0.06–6.53				0.939			0.11		0.07–16.89					0.444		3.82	0.12–118.7	
Diet 2	0.164	9.98			0.39–254.5				0.947			0.89			0.04–21.74			0.732				1.70			0.08–36.07				0.904			0.26		0.03–54.40					0.532		3.80	0.06–250.0	
Diet 3	0.800	1.45			0.08–26.14				0.019			0.08			0.03–0.90			0.735				0.65			0.05–7.88				0.227			5.13		0.36–72.68					0.590		2.64	0.08–90.03	
Diet 4	0.990	0.88			0.01–0.10				0.990			0.01			0.02–0.23			0.990				70.60			0.96–49.66				0.989			35.43		0.02–0.68					0.990		24.6	0.21–345.0	
Diet 5	0.149	10.88			0.43–277.4				0.622			2.16			0.10–46.33			0.418				3.40			0.18–65.83				0.050			1.52		0.15–0.94					0.559		3.54	0.05–246.4	
Diet 6	1 ^g^									1									1									1									1						

Diet 1, Traditional diet; Diet 2, Vegetarian diet; Diet 3, Japanese diet; Diet 4, Low energy diet; Diet 5, Healthy diet; Diet 6, Monotonous diet. ^a^ FPG increasing refers to level 0, FPG reduction between 0 and 1 mmol/L refers to level 1, reduction between 1.1 and 3 mmol/L refers to level 2, reduction between 3.1 and 5 mmol/L refers to level 3, reduction between 5.1 and 7 mmol/L refers to level 4, reduction > 7 mmol/L refers to level 5; ^b^ Comparative reference level: level 4; ^c^ Analysis adjusted for male; ^d^ Analysis adjusted for those older than 70 years old; ^e^ Analysis adjusted for those who received college or higher education; ^f^ Analysis adjusted for Group C; ^g^ Analysis adjusted for Diet 6.

## Supplementary Materials

The following are available online at http://www.mdpi.com/2072-6643/8/9/560/s1, Table S1: Structured diet for a 7-day cyclical dietary menu, Table S2: The contents of the health education, Table S3: The N-DDP score model.
