# Supplementary material for: Sustaining Effect of Intensive Nutritional Intervention Combined with Health Education on Dietary Behavior and Plasma Glucose in Type 2 Diabetes Mellitus Patients
Source: Nutrients. 2016 Sep 13;8(9):560. doi: 10.3390/nu8090560 (PMC5037545; doi:10.3390/nu8090560)
Supplement: Supplementary file 1 [file nutrients-08-00560-s001.docx]

Supplementary Materials: Sustaining Effect of Intensive Nutritional Intervention Combined with Health Education on Dietary Behavior and Plasma Glucose in Type 2 Diabetes Mellitus Patients

Rui Fan, Meihong Xu, Junbo Wang, Zhaofeng Zhang, Qihe Chen, Ye Li, Jiaojiao Gu, Xiaxia Cai, Qianying Guo, Lei Bao and Yong Li *

**Table S1.** Structured diet for a 7-day cyclical dietary menu.

| **Meal** | **Raw Food Material** | | | | | | | | | **Cooking Method** |
| --- | --- | --- | --- | --- | --- | --- | --- | --- | --- | --- |
|  | **Cereals** | **Vegetables** | **Fruits** | **Lean Meat** | **Bean Products** | **Egg** | **Milk** | **Vegetable**  **Oil** | **Salt** |  |
| Breakfast | 80 | 60 | 40 | 0 | 15 | 60 |  |  | 1 | steamed/ boiled |
| Lunch | 150 | 150 | 50 | 80 | 30 |  |  | 12 | 2.5 | Uncooked simmered/stir-fried |
| Dinner | 120 | 150 | 50 | 80 | 30 |  |  | 12 | 2.5 | Uncooked boiled/stir-fried |
| Extra food | 0 | 40 | 60 | 0 |  |  | 250 |  |  | - |
| Total | 350 | 400 | 200 | 160 | 75 | 60 | 250 | 24 | 6 | - |

Cereals (rice, wheat, black rice, corn and oat); Vegetables (bitter gourd, cabbage, tomato, green pepper, pumpkin, carrot, celery, aubergine, lettuce, spinach, wax gourd, cauliflower, cucumber, Chinese cabbage, bean sprout, onion and garlic) and fungi; Fruit (cherry tomato, apple, orange); Lean meat (pork, beef and chicken) and fish; Bean products (Tofu ,dried bean curd and soya-bean milk.

**Table S2.** The contents of the health education.

|  | **Contents** |
| --- | --- |
| Knowledge of diabetes | Symptoms of type 2 diabetes mellitus |
|  | Complications of type 2 diabetes mellitus |
| Diabetes medication | Different types of diabetic medications |
|  | The proper methods of taking diabetic medication |
| Blood glucose monitoring | The target range for blood glucose control |
|  | Methods of self-monitoring of blood glucose |
|  | Techniques of handling abnormal blood glucose |
| Healthy diet | Balanced diet for type 2 diabetics |
|  | Appropriate caloric intake for type 2 diabetics |
|  | Diet combination and cooking method for type 2 diabetics |
| Healthy lifestyl | The appropriate exercise for type 2 diabetics |
|  | Giving up bad habits and maintaining healthy behaviors |

**Table S3.** The N-DDP score model.

| **Food Items** | **N-DDP Score Model** | | | |
| --- | --- | --- | --- | --- |
|  | **C (Energy Percentage)** | **D (Rating)** | **E (Calculated Score)** | **F (Maximum Limit of Score)** |
| Cereals | 48.0 | 0.5 | 24.0 | 30.0 |
| Tubers | 2.0 | 2.0 | 4.0 | 14.0 |
| Animal products | | | | |
| Meat | 5.0 | 0.5 | 2.5 | 4.0 |
| Fish & Shrimp | 4.0 | 2.0 | 12.0 | 14.0 |
| Eggs | 2.0 | 2.0 |  |  |
| Milk | 9.0 | 1.5 | 26.5 | 27.0 |
| Beans & Products | 6.0 | 2.0 |  |  |
| Nuts & Seeds | 2.0 | 0.5 |  |  |
| Vegetables | 5.0 | 2.5 | 25.0 | 45.0 |
| Fruit | 5.0 | 2.5 |  |  |
| Pure-energy foods |  |  | 6.0 | 6.0 |
| Added fats & oils | 10.0 | 0.5 |  |  |
| Sugars & sweeteners | 1.0 | 0.5 |  |  |
| Beverages & wine | 1.0 | 0.5 |  |  |
| Total | 100.0 |  | 100.0 | 140.0 |
